# Life-threatening gastrointestinal bleeding caused by jejunal heterotopic gastric mucosa in an adult dog: a rare case report

**DOI:** 10.1186/s12917-022-03415-0

**Published:** 2022-08-16

**Authors:** Roxana Merca, Barbara Richter

**Affiliations:** 1 Department for Companion Animals and Horses, University Clinic for Small Animals, Small Animal Surgery, University of Veterinary Medicine, Veterinärplatz 1, 1210 Vienna, Austria; 2 Department for Pathobiology, Institute of Pathology, University of Veterinary Medicine, Veterinärplatz 1, 1210 Vienna, Austria

**Keywords:** Heterotopic gastric mucosa, Ectopic tissue, Anemia, Intestinal mass, Differential diagnosis, Intestinal bleeding, Case report

## Abstract

**Background:**

Heterotopic gastric mucosa has been scarcely reported in the veterinary literature. Its presence can be asymptomatic or associated with various clinical signs ranging from apathy, vomiting, to abdominal pain. This report illustrates the presence of heterotopic gastric mucosa in the jejunum of an adult dog. It is the first to describe severe anemia, requiring acute blood transfusion, following intestinal hemorrhage caused by heterotopic gastric mucosa.

**Case presentation:**

A twelve-year-old, intact male Maltese dog was presented with a history of apathy, vomiting and anemia. The dog was on a strict diet for recurrent diarrhea, food intolerance and skin allergy. Clinical examination revealed severe anemic mucous membranes and painful abdominal palpation. Blood examination confirmed severe regenerative anemia. Ultrasonography showed an intestinal neoplasm, gall bladder sludge and non-homogeneous liver parenchyma. Three-view thoracic radiographs failed to show any metastatic lesions or enlarged lymph nodes. After initial stabilization and blood transfusion, a midline exploratory laparotomy was performed. Three different masses were found in the jejunum. Resection and anastomosis of approximately 40 cm of jejunum was performed, followed by liver and lymph node biopsy and placement of an esophagostomy tube. Two days after surgery the dog started to clinically improve and was discharged from the hospital on the sixth day after surgery. Histopathology revealed the intestinal masses to be heterotopic gastric mucosa associated with intramural cystic distensions, multifocal ulceration and bleeding into the intestinal lumen. Two years after surgery, the dog did not have a recurrence of anemia or gastrointestinal signs.

**Conclusions:**

This case demonstrates that heterotopic gastric mucosa can be considered one of the differential diagnoses in case of severe anemia due to gastrointestinal hemorrhage and suspected intestinal tumors. Although in most described cases in literature the finding seems to be incidental on necropsy, our report shows that heterotopic gastric mucosa can be the etiology of life-threatening signs. In addition, because no recurrent diarrhea episodes occurred after surgical resection of the ectopic tissue, it is likely that the heterotopic gastric mucosa was the cause of the food intolerance signs in this dog.

## Background

Heterotopic mucosa is defined as morphologically normal tissue displaced in a foreign anatomical site, separated from its organ of origin and demarcated from surrounding mucosa [1]. Heterotopic gastric mucosa (HGM), gastric tissue that is found outside the stomach, has been described in human medicine in several case reports [2–14]. It has been reported in children[15] and in adults [9] with most patients being alive at the time of diagnosis. Conversely, the veterinary literature describes mostly HGM as an incidental finding during necropsy in dogs [16, 17] and cats [18].

The prevalence in human medicine is estimated to be 0.1% up to 11% [9] whilst the prevalence in veterinary medicine has been reported to be 4.1% in dogs in a single study [16]. In human medicine, HGM has been found in different locations, including esophagus [4, 9, 19, 20], duodenum [7] anorectum[8], gall bladder and cystic duct [3], airways [21], and in congenital abnormalities such as Meckel’s diverticulum and gastrointestinal duplications [22]. In contrast, HGM has been described only in the intestine of dogs and in the esophagus of cats [16–18]. In both, human and veterinary medicine, HGM is a rare finding, being mostly incidental during endoscopy or necropsy [16, 23]. Most often patients are asymptomatic on presentation, however, several cases of patients that presented minor to severe symptoms, ranging from vomiting, chronic anemia, rectal bleeding, melena, intussusception and abdominal pain have been reported [3, 7, 11–13, 15].

This case report is the first to present a life-threatening gastrointestinal hemorrhage, that required whole blood transfusion, caused by HGM in the jejunum of an adult dog. Furthermore, to the authors’ knowledge, it is the first report presenting the resolution of chronic diarrhea after removal of a jejunal HGM.

## Case presentation

A 12-year-old, client owned intact male Maltese dog was presented for further investigation of apathy, vomiting and anemia. The dog had a history of chronic diarrhea, food intolerance and atopic dermatitis, for which it was on a strict diet and permanent antiallergic therapy with oclacitinib and prednisolone. One week prior to presentation, the dog was presented at the referring veterinarian for apathy, vomiting, hyporexia and dark feces and was administered amoxicillin clavulanic acid, dexamethasone, sucralfate and vitamins. Due to lack of clinical improvement and a severe anemia (hematocrit 18.7%; range 37–55%), the dog was referred to the clinic.

On presentation, the dog was bright and alert with a body condition score of 6 out of 9 and a bodyweight of 7.8 kg. Clinical examination revealed severe anemic mucous membranes, a slightly elevated pulse rate of 124 per minute, pain signs at abdominal palpation and a cushingoid appearance. Blood examination including hematology, serum biochemistry, coagulation panel and blood type was performed (Table 1). A severe regenerative anemia (hematocrit 14.6%; range 37–55% and reticulocytes 3.2%; range 0.5–1.0%) was detected. Abdominal ultrasound scanning revealed the presence of gall bladder sludge, non-homogeneous liver parenchyma and a 2.3 × 1.8 cm neoplasm with hypoechoic areas in the jejunum. Three-view (dorso-ventral, left-lateral and right-lateral) thoracic radiographs did not show metastatic lesions or lymph nodes enlargement. The dog was administered 150 mL of DEA 1.1 negative whole blood transfusion and was scheduled for exploratory laparotomy.

**Table 1:** Results of blood examination at time of first presentation in the clinic

	Patient value	Laboratory reference interval
Erythrocytes (10^6^/µl)	**2.84**	5.50 – 8.00
Hemoglobin (g/dl)	**4.0**	12 – 18.0
Hematocrit (%)	**14.6**	37.00 – 55.00
MCV (fl)	**51.4**	60.0 – 77.0
MCH (pg)	**14.1**	19.0 – 24.5
MCHC (g/dl)	**27.4**	31.0 – 34.0
RDW (%)	**3.2**	0.5 – 1.0
RDW absolute ( /µl)	**89460.0**	> 60000.0
Platelet count (10^3^/µl)	**693**	150 – 500
WBC (/µl)	**37990.0**	6000.0 – 15000.0
Neutrophils (%)	**85.8**	55.0 – 75.0
Lymphocytes (%)	**6.6**	13.0 – 30.0
Monocytes (%)	**6.4**	< 5.0
Eosinophils (%)	0.7	< 4.0
Basophile (%)	0.2	< 1.0
Absolute neutrophil count (/µl)	**32595.42**	3300.00 – 11250.00
PTT (sec.)	14.0	8.0 – 15.0
PT (sec.)	**7.1**	8.0 – 10.0
TT (sec.)	13.6	< 20.0
Glucose (mg/dl)	**115.0**	55.0 – 90.0
Urea (mg/dl)	38.1	20.0 – 40.0
Creatinine (mg/dl)	0.70	0.40 – 1.20
Total protein (g/dl)	6.62	6.00 – 7.50
Albumin (g/dl)	2.89	2.58 – 4.73
Alkaline phosphatase (U/L)	15	< 130
ALT (U/L)	24	< 80
Bilirubin (mg/dl)	0.01	< 0.80
Lipase (U/L)	**223**	< 125
Sodium (mmol/L)	145	140 – 152
Potassium (mmol/L)	**3.3**	3.6 – 5.6
Chloride (mmol/L)	108	95 – 113
Phosphorus (mmol/L)	**0.81**	0.90 – 1.60
C-reactive protein (mg/L)	10.0	< 35.0
Cobalamin (Vit B12) (pg/ml)	**936**	300–800
Blood group	DEA 1.1 negative	

Parameters outside the reference interval are highlighted in bold

The dog was premedicated with methadone 0.2 mg/kg intravenously (IV). General anesthesia was induced with fentanyl (5 µg/kg IV), midazolam (0.2 mg/kg IV) and propofol titrated to effect until tracheal intubation was achieved. Anesthesia was maintained with isoflurane (end-tidal 1.0 – 1.2 vol%) in 100% oxygen through a circle rebreathing system. Additionally, a fentanyl constant rate infusion (CRI) (1.5 – 2 µg/kg/h IV) was administered throughout the procedure. Amoxicillin clavulanic acid (22 mg/kg IV) was administered during anesthesia induction and every 90 min during the surgical procedure.

An exploratory median celiotomy was performed, which revealed three different masses arising from the jejunum, all of them intimately associated with the intestine. Two of them were approximately 2 × 2 cm, were only a few centimeters apart, had a cyst-like appearance and involved the caudal part of the jejunum. In addition, there was a mass in the mid-jejunum, approximately 1 × 2 cm in size, with a very similar appearance. Resection and anastomosis of approximately 40 cm of the jejunum was performed. Additionally, a 1 × 1 cm mass associated with the caudate process of the liver was resected using a guillotine technique. A wedge biopsy was taken from the enlarged mesenterial lymph nodes and all the specimens were sent for histopathologic examination. No other macroscopic changes and no peritoneal effusion were seen intraabdominally. Abdominal lavage with Ringer’s solution and omentalization of the anastomosis site were performed prior to abdominal closure. An esophagostomy feeding tube was placed at the end of the procedure.

The dog recovered uneventfully from general anesthesia and was administered methadone 0.2 mg/kg every 4 h, lidocaine CRI (50 µg/kg/min) and metamizole 25 mg/kg. The hematocrit on the first postoperative day increased to 32%. Despite the planned analgesic treatment, the patient showed signs of pain during the early recovery period and therefore, a CRI of methadone, lidocaine and ketamine (1–2 ml/kg/h) was initiated. Additional postoperative therapy included crystalloid fluid therapy with added potassium at 3 ml/kg/h (IV), esomeprazole 1 mg/kg (IV), sucralfate 30 mg/kg per os (po), maropitant 1 mg/kg (IV), ursodeoxycholic acid 7.5 mg/kg and S-adenosyl-methionine 25 mg/kg (po). In order to minimize intraabdominal pressure, the esophageal feeding tube was advanced into the stomach and all fluid and gas that had accumulated secondary to the postoperative paralytic ileus was retrieved through the tube. The dog was alternatively fed via the feeding tube and gastric contents were aspirated when percussion of the cranial abdomen revealed gastric distention, every 2 to 6 h. On the second postoperative day, analgesic treatment was changed to buprenorphine 20 µg/kg every 6 h and lidocaine CRI 40 µg/kg/min. The need for aspiration of the stomach decreased considerably and therefore, on the third postoperative day, the esophageal feeding tube was retrieved into the esophagus and only used for feeding. The dog started defecating on the second day postoperatively and retained all the food. The dog was discharged on the sixth postoperative day due to improvement of clinical condition. Two days follow-up showed proper wound healing and presented no signs of abdominal pain.

**Fig. 1:**
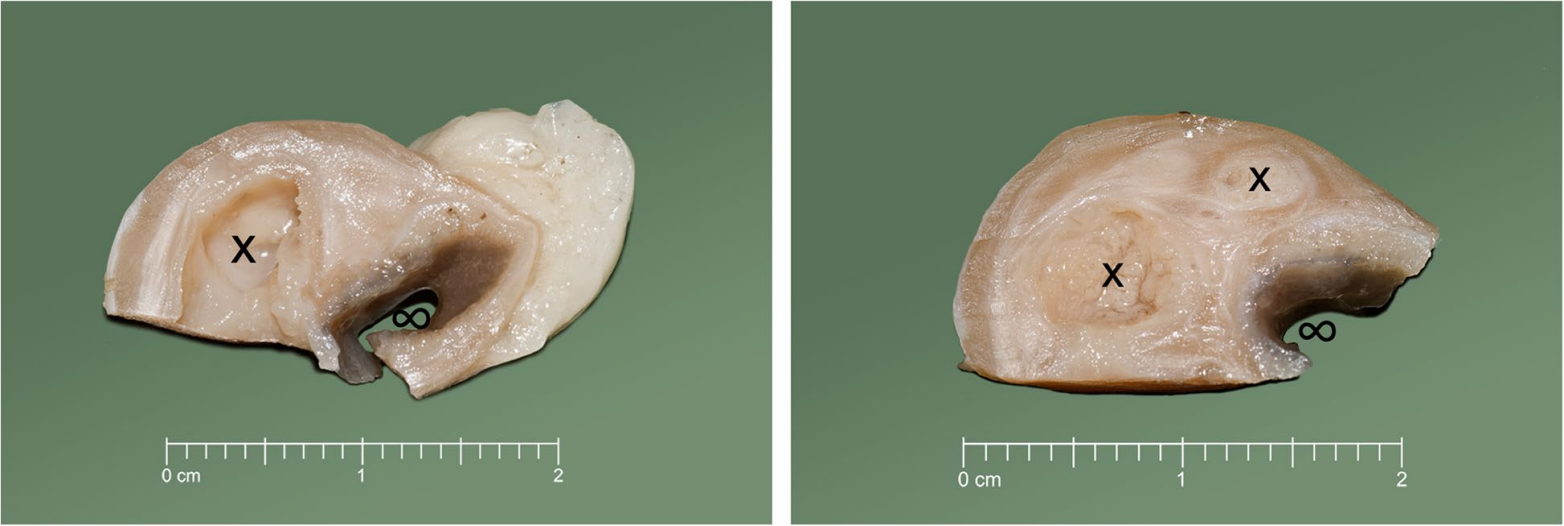
Formalin-fixed and trimmed intestinal specimen. Multiple cysts (x) expand the intestinal wall. To the right the intestinal lumen is seen (∞)

**Fig. 2:**
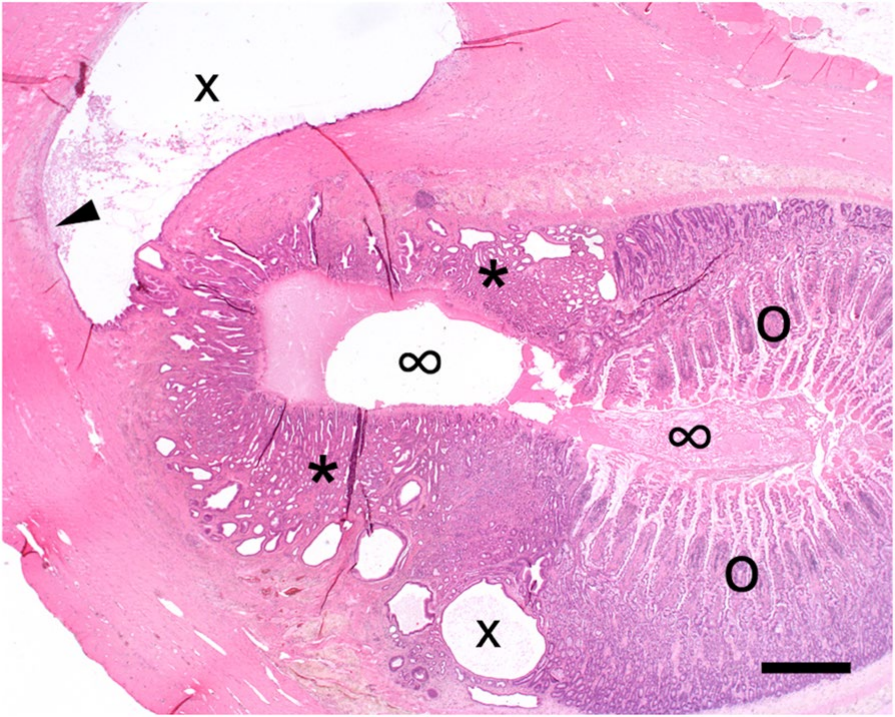
Histological cross-section of intestinal mass. The normal jejunal mucosa on the right side (o) encircles the intestinal lumen (∞) and is partly displaced by heterotopic gastric fundic mucosa (*) extending through the submucosa into the muscularis. The cystic spaces (x) show focal ulceration (arrowhead) with extravasation of inflammatory cells and erythrocytes. Hematoxylin–Eosin, bar = 1 mm

**Fig. 3:**
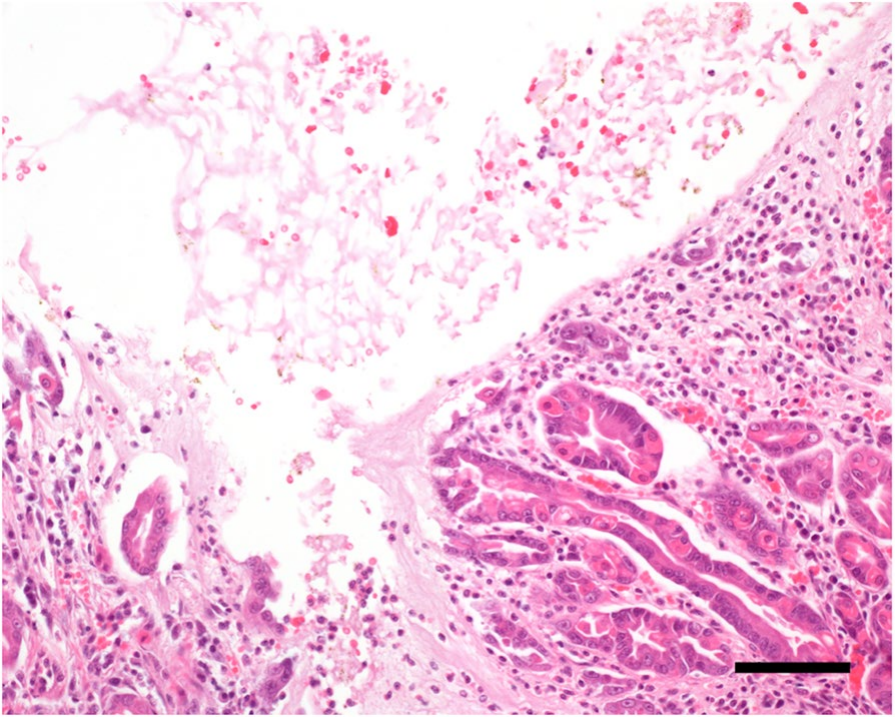
Area of heterotopic gastric mucosa showing unorganized arrangement of the different epithelial cell types and ulceration accompanied by acute bleeding into the intestinal lumen, hyperemia and neutrophilic and lymphoplasmacellular infiltration. Hematoxylin–Eosin, bar = 80 µm

Histopathological examination of the three retrieved jejunal masses revealed a similar histological picture. The jejunal wall was expanded at the antimesenterial and lateral parts by cysts of 1.5 cm of diameter (Fig. 1). In these areas, the jejunal mucosa was replaced by fundic gastric mucosa including faveolar, mucus neck, chief and parietal cells. These cells formed pit-like structures but often without proper arrangement. The more basal parts extended into the submucosa and muscularis and mostly faveolar cells covered the cystic spaces, which resulted in the increased diameter of the intestine (Fig. 2). The lining epithelium ranged from columnar to flattened cells and was moderately activated. It was mostly arranged as a single layer but focally it formed few micropapillary and small papillary extensions into the cyst lumen. Frank atypia was not noted, and the mitotic rate did not seem to be increased. Multifocally, the gastric epithelium was ulcerated including acute lesions such as bleeding into the gut or cyst lumina and diffuse neutrophilic infiltration (Fig. 3) as well as more chronic lesions like granulation tissue formation with fibroplasia, infiltration with plasma cells, lymphocytes and foamy or few hemosiderin-laden macrophages. The lumina of cysts communicated with the intestinal lumen. The cyst lumina contained proteinaceous fluid, foamy macrophages, neutrophils and extravasated erythrocytes. The lymph vessels in the surrounding muscularis were dilated.

The adjacent jejunal mucosa showed a slightly increased eosinophilic infiltration in the lamina propria. The intestinal lymph node was infiltrated by moderate numbers of eosinophils and few neutrophils and hemosiderin-laden macrophages. The liver exhibited a moderate steroid hepatopathy, minor intracellular cholestasis in centrolobular areas and few periportal neutrophils as well as a 1 cm-sized nodular hyperplasia.

While being in home care, the dog was constantly improving, started to eat but was additionally fed over the feeding tube by the owner when the energy requirements were not reached. Because the dog refused to eat his usual hypoallergenic diet, the owner was advised to feed any commercial diet that was preferred by the dog. After changing to a commercial food, the dog started to eat normally. The dog defecated daily and normally, without signs of melena or diarrhea. No other abnormal gastrointestinal signs were present. A few days afterwards, the esophageal tube was removed. Two-month follow up period, which included three clinical controls at the referring veterinarian, showed no clinical deterioration or recurrent diarrhea. Two years after the surgery, and although the owner just partially changed the diet to the one administered before the intervention, the dog was still in good clinical condition without showing recurrence of diarrhea.

## Discussion and conclusions

This is the first report of a life-threatening intestinal hemorrhage caused by HGM in the intestine of an adult dog. Additionally, to the authors’ knowledge, this is the first case report describing the resolution of chronic diarrhea, after resection of intestinal HGM.

The dog presented in our case report had signs of acute vomiting, anorexia, abdominal pain and severe anemia. Gastrointestinal signs are one of the most common reasons for clinical presentation [24]. Differential diagnoses for the acute onset of these clinical signs include gastroenteritis [25], gastrointestinal ileus [25], foreign bodies [25, 26], gastrointestinal tumors [25], gastrointestinal ulceration [25], gastrointestinal perforation or abscesses [25], pancreatitis [24], cholecystitis [27], cholelithiasis [28] and tumors of the pancreas or the biliary system [25]. Anemia can be presented in correlation to gastrointestinal signs or as a comorbidity[29]. When presented as a correlation to gastrointestinal signs, it is mostly related to gastrointestinal bleeding [29, 30]. Differential diagnoses for gastrointestinal bleeding include gastroenteritis [29], gastrointestinal ulcerations [31] and gastrointestinal neoplasms [32].

Tobleman and Sinnott [33] described a case of a 7-month-old dog with a congenital malformation of HGM with secondary bacterial infection located in the mid-jejunum, and although this dog presented a severe clinical condition, gastrointestinal signs were not present. A very severe case of gastrointestinal bleeding, leading to death, of a 7-year-old boy was described by Lambert et al. [34]. The child had several hospital admissions for unexplained massive, life-threatening gastrointestinal hemorrhage. The source of bleeding was never identified, despite multiple times work up, and negative Meckel’s scan. On the last presentation, clinical examination revealed abdominal pain, non-bloody-diarrhea and vomiting. On work-up, intestinal perforation was diagnosed and after laparotomy with primary closure of perforation and resection of an adjacent segment of abnormal-appearing bowel, the patient died within 24 h from overwhelming sepsis. The pathologic report of the resected small bowel segment removed prior to death showed gastric mucosa of fundic type but merged with foci of small intestinal mucosa. Like in the previously described case, our patient was presented with painful abdomen and vomiting. In both cases gastrointestinal problems occurred before, but further work-up did not find the cause. Also, the life-threatening gastrointestinal bleeding was caused by HGM located in the intestine, and for both cases there were three different localizations showing pathological findings. However, although in the case described by Lambert et al. the three large areas found during the autopsy were consistent with gastric fundic mucosa, there was no evidence of ulceration or acute inflammation [34]. Contrary to the previously described patient, in our case, there was no perforation of the intestine and no signs of septic peritonitis or likelihood of it progressing to sepsis. Nevertheless, the dog in our report had a life-threatening clinical presentation demanding an aggressive therapy, since the clinical deterioration and anemia were rapidly progressing.

Several publications described intestinal neoplasms in dogs [35–37] and their outcome. Owners and veterinarians should be aware of the possible differential diagnosis of changes like HGM when discussing treatment options. Given the fact that the dog in our case report was treated for food intolerance before surgery, and that up to two years after the surgical resection of HGM the dog did not present diarrhea or other gastrointestinal signs, it is likely that HGM could have been the etiology of these signs. Food intolerance has been described in dogs and cats before, but the mechanism remains undetermined, and the lack of specific testing and the variability of clinical signs have shown that our understanding is limited [38]. Our report shows that ultrasonographic tumor-like intestinal changes, although accompanied with severe clinical signs, can have a good prognosis.

One of the main difficulties in diagnosing HGM is the lack of a specific test. Different methods have been described but they have variable sensitivity and specificity. In our case, ultrasonography was sensitive in diagnosing the intestinal mass but lacked in specificity for the final diagnose. Blood examinations showed unspecific findings: increased white blood cells count showed an unspecific inflammatory process; decreased erythrocytes, hematocrit and hemoglobin and elevated reticulocytes showed an unspecific regenerative anemia, increased lipase enzymes without increase in liver enzymes could have been related to the gall bladder sludge, increased platelet cells and decreased prothrombin time could have been related to the intestinal bleeding. Therefore, blood examination did not show any specific findings that could have been related to HGM.

In human medicine, endoscopy seems to be one of the most sensitive methods for esophageal and rectal HGM [2, 23] and, for intestinal HGM, capsule endoscopy has been successfully used to localize the lesion prior to surgery [3, 13]. In veterinary medicine, since HGM is a rare incidental finding, necropsy is the most commonly described diagnostic method [16, 18]. However, with constant improvement in treatment options and increasing compliance of owners, some symptomatic cases with heterotopia have been found in the living animal [33, 39]. In these cases, the final diagnosis was made by histopathological examination of resected specimens. Nevertheless, capsule endoscopy has been also used in dogs, and the site of gastrointestinal bleeding could be localized in 24 out of 39 recordings [40]. Unfortunately, the final diagnosis brought by biopsy, is currently not technically available with capsule endoscopy.

Described HGM in dogs often consists of several epithelial cell types, corresponding to normal gastric mucosa [16, 17, 33]. These could also extend into the submucosa [16], which has been also reported in humans [2]. They also have been associated with diverticular lesions of the intestine [33, 41]. Terada [9] hypothesized that HGM made up of several cell types is more likely of congenital origin representing a malformation, in contrast to areas of purely foveolar gastric cells inside the intestinal mucosa, which could rather be the result of metaplasia. Thus, it is likely that in the case presented here, the lesions were of congenital origin. Although the expansion of the HGM into the submucosa and muscularis resulted in extensive cystic expansion of the intestinal wall, the histological picture of the heterotopic cells does not support a neoplastic process as described by Panigrahi et al. [17]. The cells did show disorderly arrangement and some activation but no frank atypia, invasive growth or increased mitotic rate. Also, a simultaneous malignant transformation of different cell types at three unconnected areas of HGM seems unlikely.

In conclusion, the authors support histopathological examination of all resected specimens of intestine, regardless of the macroscopic appearance, for a proper assessment of the prognosis and further treatment options. Based on this case report, we would recommend including heterotopic gastric mucosa as one of the differential diagnoses for gastrointestinal symptoms, regardless of their severity. Because no recurrent diarrhea episodes occurred after surgical resection of the ectopic tissue, it is likely that the heterotopic gastric mucosa was the cause of the food intolerance signs in this dog.

## Data Availability

All available data are within this paper.

## Abbreviations

CRI: Constant rate infusion
HGM: Heterotopic gastric mucosa
IV: Intravenously
Po: Per os
